# Parenteral CHIKV mRNA Vaccination Reshapes the Gut Microbial, Metabolic, and Immune Landscapes

**DOI:** 10.1002/mco2.71138

**Published:** 2026-11-08

**Authors:** Hongyu Chen, Wenhao Xie, Yun Yang, Cong Tang, Xiaoming Liang, Yuxia Yuan, Junbin Wang, Yanan Zhou, Hao Yang, Qing Huang, Wenqi Quan, Wenhai Yu, Haixuan Wang, Daoju Wu, Youchun Wang, Shuaiyao Lu

**Affiliations:** ^1^ State Key Laboratory of Respiratory Health and Multimorbidity Institute of Medical Biology, Chinese Academy of Medical Sciences & Peking Union Medical College (IMBCAMS&PUMC) Kunming Yunnan China; ^2^ Key Laboratory of Pathogen Infection Prevention and Control (Peking Union Medical College) Ministry of Education Beijing China; ^3^ Yunnan Key Laboratory of Cross‐Border Infectious Disease Control and Prevention and Novel Drug Development Kunming China

**Keywords:** CHIKV, gut microbiota, immune modulation, NHP model, *Segatella copri*, vaccine

## Abstract

The gut microbiota is increasingly recognized as a critical modulator of vaccine efficacy. However, how parenteral vaccines—particularly those against non‐enteric viruses—reshape the intestinal microenvironment remains poorly defined. Here, we investigated the impact of two validated CHIKV mRNA vaccines on gut microbial composition, metabolic activity, and immune features in rhesus macaques. Using a longitudinal multi‐omics approach, including metagenomics, metabolomics, and proteomics, and comparing with CHIKV‐infected animals, we assessed whether systemic vaccination alone, in the absence of intestinal viral replication, was associated with changes in the gut microenvironment. We demonstrate that vaccination induces reproducible shifts in gut microbiota composition, notably enriching *Segatella copri*, similar to infection‐associated changes. These alterations are accompanied by remodeling of purine and bile acid metabolism, with persistent increases in xanthine and uric acid. Upon viral challenge, vaccinated animals exhibit reduced intestinal viral burden, attenuated pathology, and elevated expression of proteins related to antigen presentation, immunoglobulins, and epithelial barrier function. These findings provide direct evidence that systemic CHIKV mRNA vaccination is associated with changes in the gut microbial, metabolic, and immune landscapes, highlighting the gastrointestinal tract as an active and responsive target of parenteral vaccinations.

## Graphical abstract

This study provides multi‐omics evidence that parenteral mRNA vaccination against CHIKV, even in the absence of viral infection, can profoundly remodel the host intestinal ecosystem—recapitulating key microbial, metabolic, and GI immune features typically associated with active CHIKV infection, characterized by enrichment of *Segatella copri*, activation of purine and bile acid pathways, and increased xanthine and uric acid levels. These changes are tightly linked to microbial restructuring and immune activation. Upon challenge, vaccinated animals display reduced intestinal viral burden, attenuated pathology, and enhanced antigen presentation, immunoglobulin responses, and epithelial barrier function. Together, these findings identify the gut as a responsive target of parenteral vaccination and reveal antigen‐driven microbial–immune crosstalk as a key mechanism shaping vaccine‐induced host responses.

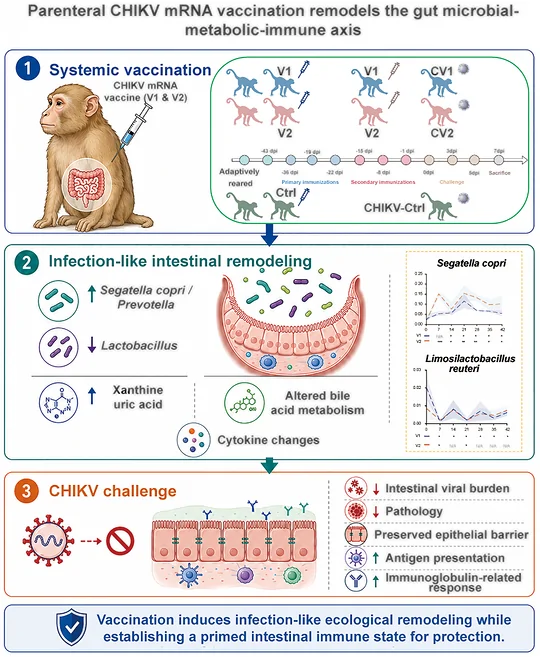

## 1 Introduction

Chikungunya virus (CHIKV), a highly pathogenic mosquito‐borne alphavirus [1], poses a growing global health threat, particularly in tropical and subtropical regions [2, 3]. While acute infection typically manifests as febrile illness with polyarthritis, a substantial proportion of patients develop chronic arthralgia and persistent inflammatory sequelae closely resembling rheumatoid arthritis (RA) [4]. Importantly, studies in both murine and non‐human primate (NHP) models revealed that CHIKV infection—a non‐enteric viral challenge—can induce marked alterations of gut microbial composition, which in turn exacerbates the disease [5]. These findings point to a previously underappreciated role for the gut as both a responder and a regulator during systemic CHIKV infection. Beyond nutrient absorption and immune surveillance, the gastrointestinal (GI) tract is increasingly recognized as a dynamic interface where microbial, metabolic, and immune networks converge to shape systemic health [6, 7]. Within this complex ecosystem, the gut microbiota and their derived metabolites profoundly influence the development, function, and regulation of the immune system—effects extending well beyond the mucosal surface [8]. Conversely, systemic immune perturbations—such as infection and inflammation—also reshape the composition and function of the gut microenvironment [9, 10]. Understanding this bidirectional crosstalk is critical for optimizing immunological interventions in health and disease.

Although many studies have explored how enteric infections disrupt gut microbial homeostasis [11], few have investigated how non‐enteric infection events—such as systemic vaccination—impact the intestinal ecosystem, that is, whether vaccine‐induced immunity can reprogram this microbiota‐immune axis. Most current vaccines are evaluated primarily based on systemic immune indicators—such as serum antibodies, peripheral T‐cell responses, and protection rates—without fully considering the broader physiological context of immune activation [12, 13]. Yet the gut harbors a dense population of immune cells, is richly vascularized, and is constantly exposed to microbial and dietary antigens [7]. Thus, we hypothesized that vaccine‐induced immune responses, even when initiated at distant sites, may influence intestinal physiology, alter host‐microbe interactions, and modulate the GI immune landscape. Notably, Feng et al. demonstrated that antibiotic‐mediated disruption of the gut microbiota affected both the magnitude and durability of primary and secondary immune responses to rabies vaccination in humans, mediated in part by microbial metabolites such as secondary bile acids [14]. These findings underscore the gut microbiome's critical role in shaping vaccine‐induced immunity.

Accordingly, we employed two previously validated CHIKV mRNA vaccines, V1 and V2. Our previous efficacy studies demonstrated that both vaccines provided effective protection against CHIKV infection in mice, rhesus macaques, and a lethal AG129 mouse model. However, V2 exhibited greater protective efficacy than V1 and induced more sustained B‐cell activation, a stronger IgG‐dominated memory response, and a more robust recall response following viral challenge [15]. These distinct characteristics provided a unique framework for investigating whether vaccine‐induced alterations in the gut microenvironment are associated with differences in vaccine immunogenicity and protective efficacy, and whether vaccines with distinct immune profiles nevertheless elicit shared changes in microbial community structure and metabolic pathways.

In a NHP model, we collected fecal and blood samples before and after immunization and following CHIKV challenge, with unvaccinated infected animals as controls. Through integrated multi‐omics analyses, including metagenomics, metabolomics, and circulating cytokine profiling, we systematically characterized the dynamic responses of the intestinal microenvironment to vaccination. Intestinal tissues collected after challenge were further subjected to proteomic analysis to define local immune and epithelial responses in the context of vaccine‐induced protection (Figure 1A). Furthermore, by integrating cross‐sectional comparisons with longitudinal comparisons, we aimed to determine whether CHIKV mRNA vaccination could alter the intestinal microbial, metabolic, and immune microenvironment. More importantly, this comparative framework allowed us to determine whether these vaccination‐associated alterations were concordant with, or distinct from, those induced by active CHIKV infection. To our knowledge, this is the first comprehensive multi‐omics investigation of vaccination‐associated intestinal remodeling in a NHP model incorporating live‐virus challenge. These findings expand our understanding of vaccine–gut interactions and may inform the evaluation and rational design of future vaccines.

**FIGURE 1:**
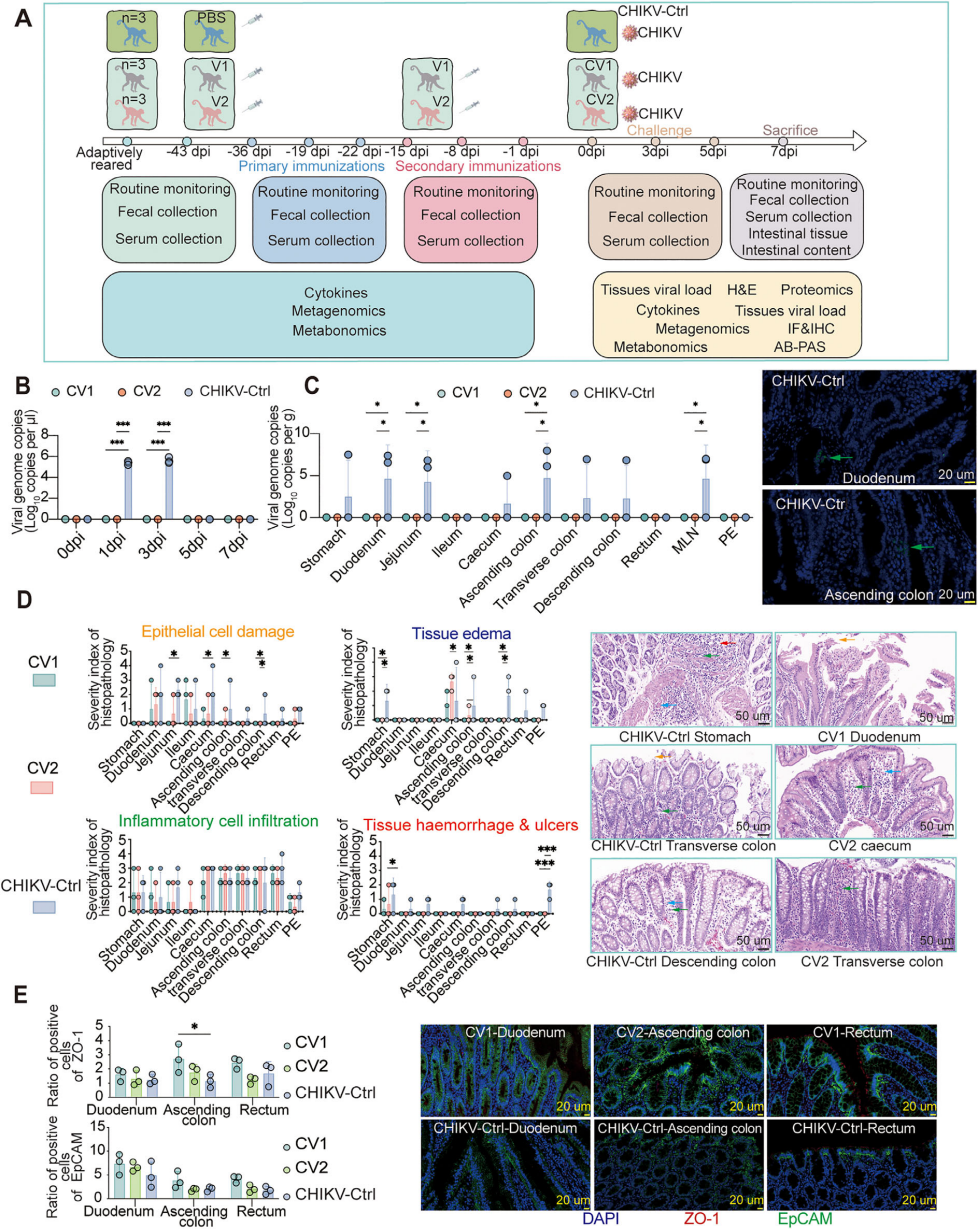
Evaluation of gastrointestinal protection by CHIKV mRNA vaccination in rhesus macaques. (A) Nine rhesus macaques were randomly assigned to vaccine group 1, vaccine group 2, and the control group. Two rounds of immunization were conducted, with continuous collection of fresh fecal and serum samples before and after vaccination. At the end of the second vaccination, the animals were intramuscularly challenged with CHIKV and monitored for 7 days. Afterward, all animals were euthanized to collect gastrointestinal contents and tissues for assessing the viral load, histopathology, proteomics, metagenomics, and metabolomics. See the “Methods” section for more details. This schematic was created using FigDraw (license: SYUTYcc079). CHIKV genomic RNA levels in the blood (B) and gastrointestinal tissues (C) (left panel) were quantified by qRT‐PCR targeting the E1 gene, with representative immunofluorescence staining for viral presence in intestinal tissues shown in the right panel. (D) The histopathological severity index (left) was based on tissue lesion scoring. Representative H&E‐stained images are shown (right). (E) Immunofluorescence staining of epithelial tight junction markers ZO‐1 and EpCAM in gut tissues (left panel). Quantification was performed using ImageJ. Representative images are shown (right panel). The statistical significance of differences was analyzed using one‐way ANOVA and post hoc Scheffe's test with FDR correction (*q < 0.05).

## 2 Results

### 2.1 CHIKV Vaccination Alleviated GI Damage and Supported Epithelial Tight Junction Integrity

Both CHIKV mRNA vaccines had previously been validated for cross‐lineage protective efficacy. Consistent with earlier findings, vaccination reduced systemic viral loads and significantly protected the GI tract after infection [15]. Following challenge, intestinal viral RNA was undetectable in vaccinated animals (CV1 and CV2), whereas CHIKV‐Ctrl animals retained high intestinal viral burdens at 7 dpi (Figure 1B,C). Histopathology showed that vaccination mitigated CHIKV‐induced GI injury, reducing epithelial cell damage and tissue edema (Figure 1D). Both groups nevertheless showed comparable intestinal inflammatory cell infiltration after infection, indicating persistent immune activation independent of overt tissue injury. Multiplex immunofluorescence staining and quantification of epithelial tight junction markers ZO‐1 and EpCAM revealed significantly higher expression and continuity in vaccinated animals, supporting a protective effect of vaccination on epithelial integrity under viral challenge (Figure 1E). Together, these findings confirmed that CHIKV mRNA vaccination prevented systemic and intestinal viral replication, attenuated GI pathology, and preserved key structural components of the gut epithelial barrier.

### 2.2 CHIKV mRNA Vaccination Induced an Infection‐Like Gut Ecological State

To explore how CHIKV vaccination affected the intestinal microenvironment, we longitudinally profiled fecal samples from NHPs before and after two rounds of CHIKV mRNA vaccination (S1: primary immunizations; S2: secondary immunizations). Metagenomic sequencing revealed reduced microbial diversity and richness post‐immunization, particularly in V2_S1 (Figure 2A, Figure S1A). Principal coordinate analysis (PCoA) further demonstrated that vaccination alone induced significant compositional shifts, resulting in distinct microbial clusters (Figure 2B), and that a single dose was sufficient to alter the microbial community structure (Figure S1B). The post‐vaccination microbial profile more closely resembled that of the CHIKV‐infected group (C) than the pre‐vaccination baseline (Figure S1C). At the phylum level, immunization significantly suppressed Bacillota while enriching Bacteroidota and Spirochaetota, mirroring infection‐driven (CHIKV‐Ctrl) trends (Figure 2C–E). Genus‐level analyses showed depletion of *Lactobacillus johnsonii* and *Limosilactobacillus reuteri*, with concurrent enrichment of *Segatella copri (formerly Prevotella copri)* and *Treponema* sp. in both vaccine groups (particularly in V2, where their abundances increased significantly as early as 7 days after immunization compared with Ctrl) (Figure 2F, Figure S1D). These shifts in relative microbial abundance were similar to those observed in CHIKV‐Ctrl animals (Figure S1E,F), suggesting that vaccination‐associated changes resembled some features of infection. Given the central role of inflammation in CHIKV‐induced intestinal alterations, we profiled serum immunoregulatory and pro‐inflammatory cytokines following vaccination (Figure S2A,B). Both vaccines (V1 and V2) induced robust cytokine responses, with notable upregulation of IL‐17A, IL‐18, MIP‐1α/β, and MCP‐1, broadly similar to expression patterns observed in unimmunized‐infected animals (CHIKV‐Ctrl). Interestingly, cytokines such as TGF‐α and CD40L—associated with Treg induction and antigen presentation—were induced by vaccination and exhibited rapid upregulation following viral challenge. They exhibited transient upregulation after the first immunization, which waned and was subsequently reactivated upon the second dose. The V2 vaccine elicited stronger activation of IL‐17A, IL‐18, IL‐10, and MCP‐1 following immunization (Figure S2B). We next challenged vaccinated animals intramuscularly with CHIKV and longitudinally tracked fecal microbiota to evaluate vaccine‐induced alterations of gut microbial composition. PCoA revealed no significant differences (*p* > 0.05) in gut microbiota composition before and after challenge in either vaccine group (CV1 and CV2) (Figure 3A, Figure S3A,B), nor did these profiles cluster away from those of the unimmunized‐infected group (CHIKV‐Ctrl). Alpha diversity indices and taxonomic composition were similarly unaffected (Figure S3C,D), with major phyla—including Spirochaetota, Bacteroidota, and Bacillota—remaining at infection levels (Figure S3E). At the genus and species level, *L. johnsonii* significantly declined post‐challenge, while *Segatella copri*, *Prevotella* sp., and *Treponema* sp. remained unchanged and comparable to CHIKV‐Ctrl (Figure 3B). Heatmaps showed that probiotic species—especially *Lactobacillus* spp.—were consistently reduced after CHIKV challenge in both vaccinated and CHIKV‐Ctrl groups, suggesting that prior immunization did not protect against this microbial perturbation (Figure 3C). Functional annotation based on the Virulence Factor Database (VFDB) revealed that *Prevotella* and *Segatella* exhibited a high cumulative contribution to virulence‐associated gene profiles (Figure S3F). To further validate these results, we examined the microbiota in both the small and large intestines. Post‐challenge, microbial alpha diversity in both regions showed no significant difference between vaccinated and CHIKV‐Ctrl groups (Figure S4A). Consistently, PCoA revealed convergence of gut microbiota profiles across vaccinated and CHIKV‐Ctrl groups in the small intestine (Figure 3D, Figure S4B). Taxonomic analyses confirmed that *Segatella copri* and *Prevotella* sp. were dominant in both the small and large intestines across CHIKV‐Ctrl and challenged vaccinated animals (CV1 and CV2) (Figure 3E, Figure S4C).

**FIGURE 2:**
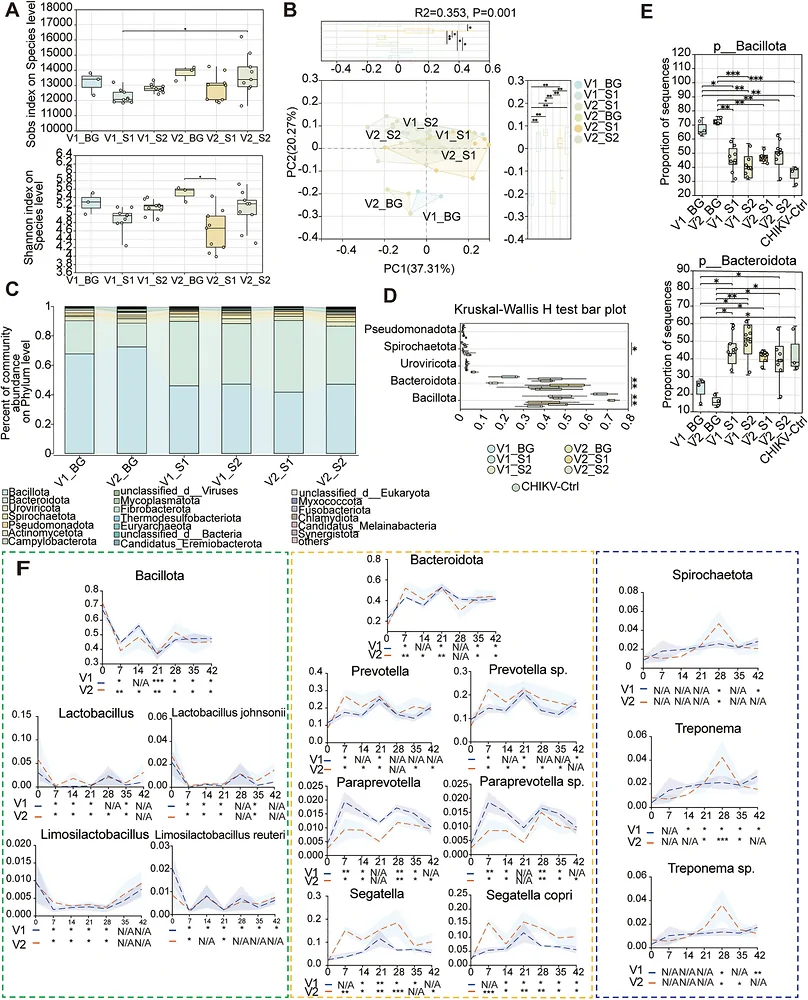
Shifts in fecal microbiota following CHIKV mRNA vaccination. (A) Alpha diversity indices of fecal microbiota before and after vaccination: Sobs (top panel) and Shannon (bottom panel). The Friedman test was used, followed by pairwise Wilcoxon signed‐rank tests with FDR correction (*q < 0.05, **q < 0.01, ***q < 0.005). (B) Principal coordinate analysis (PCoA) of fecal microbiota based on metagenomic sequencing at baseline (BG) and after each vaccination (S1, S2). Statistical differences among clusters were analyzed using permutational analysis of variance (PERMANOVA; 999 permutations). *R* ^2^ was used to interpret the degree of difference among groups. The threshold for statistical significance was set at *p* < 0.05. (C) Relative abundance of bacterial phyla visualized using bar plots. (D) The top five most abundant phyla were selected for statistical analysis (Wilcoxon rank‐sum test with FDR correction; *q < 0.05, **q < 0.01). (E) Differences in the abundance of each phylum among vaccinated (V), vaccinated‐infected (CV), and CHIKV‐Ctrl (C) groups were compared (Kruskal–Wallis test (Dunn's post hoc) with FDR; *q < 0.05, **q < 0.01, ***q < 0.005). (F) Temporal dynamics of genera and species corresponding to significantly altered bacterial phyla compared with their abundance at 0 dpi. The Friedman test was used, followed by pairwise Wilcoxon signed‐rank tests with FDR correction (*q < 0.05, **q < 0.01, ***q < 0.005).

**FIGURE 3:**
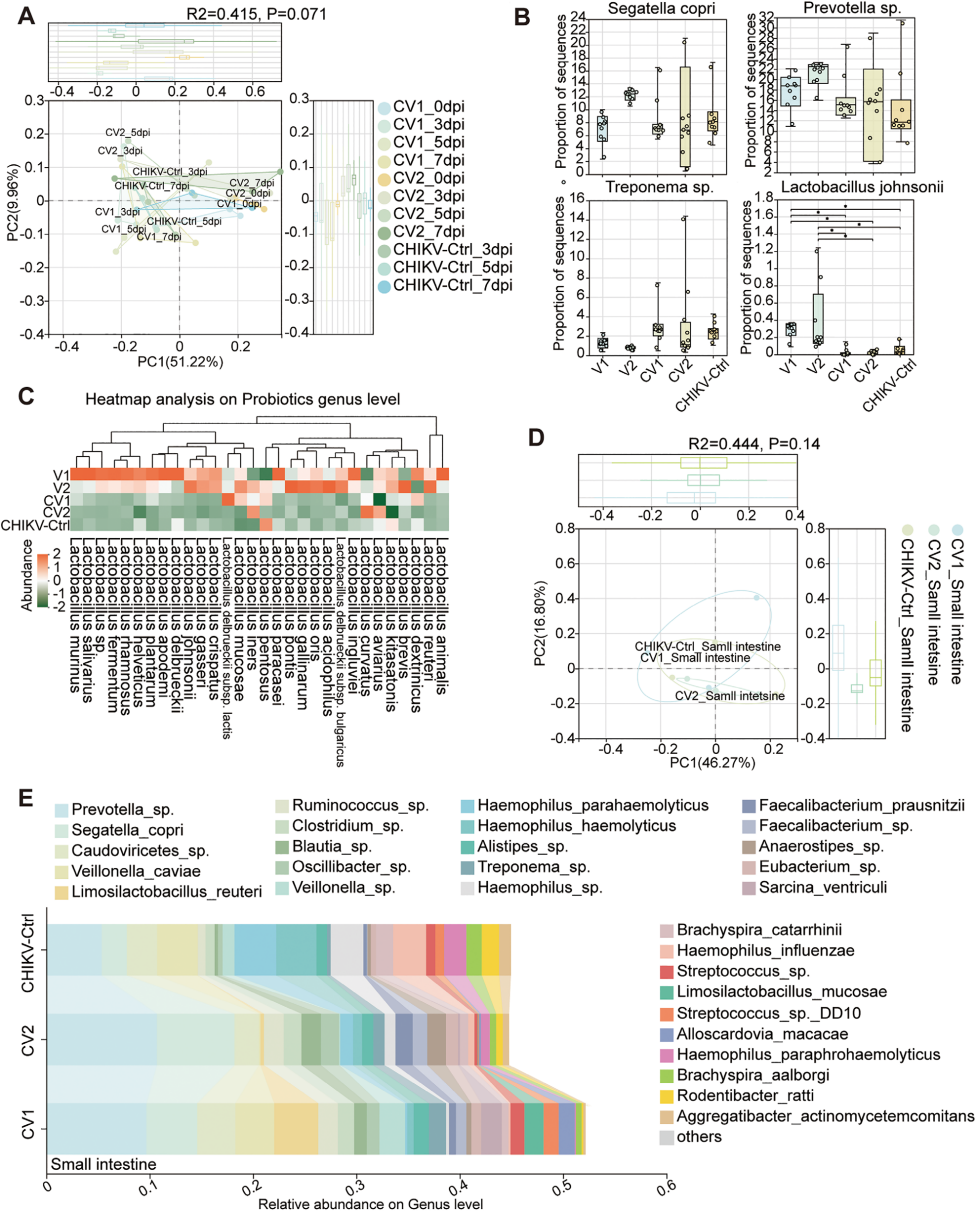
Characteristics of fecal microbiota after infection in vaccinated and CHIKV‐infected animals. (A) PCoA of fecal microbiota based on metagenomic sequencing for the vaccinated (0 dpi), vaccinated‐infected (CV1&2_3dpi, CV1&2_5dpi, CV1&2_7dpi), and CHIKV‐Ctrl (C_3dpi, C_5dpi, C_7dpi) groups. Statistical differences among clusters were analyzed using permutational analysis of variance (PERMANOVA; 999 permutations). *R* ^2^ was used to interpret the degree of difference among groups. The threshold for statistical significance was set at *p* < 0.05. (B) Comparative abundance of key microbes in the V, CV, and CHIKV‐Ctrl groups (Kruskal–Wallis test followed by Dunn's multiple‐comparison test with FDR correction; *q < 0.05). (C) Heatmap showing the abundance of the probiotic genus *Lactobacillus* across groups. (D) PCoA of small intestinal microbes from the vaccinated‐infected (CV) and infected (CHIKV‐Ctrl) groups. Statistical differences among clusters were analyzed using PERMANOVA (999 permutations). *R* ^2^ was used to interpret the degree of difference among groups. The threshold for statistical significance was set at *p* < 0.05. (E) Composition and structure of the small intestinal microbiota (genus level) in both groups.

Taken together, both V1 and V2 induced broadly similar intestinal microbial remodeling before challenge, including reduced microbial diversity, depletion of *Lactobacillus* spp., and enrichment of *Segatella copri* and *Treponema* spp. However, these changes were more pronounced in V2, with stronger induction of IL‐17A, IL‐18, IL‐10, and MCP‐1. Following CHIKV challenge, the microbiota of both vaccinated groups remained comparable to that of unvaccinated infected animals. Thus, despite differences in immunogenicity and protective efficacy, the two vaccines elicited largely shared patterns of intestinal remodeling.

### 2.3 CHIKV mRNA Vaccination Induced Coordinated Remodeling of the Gut Microbiota and of Purine and Bile Acid Metabolism

To determine how vaccination‐associated shifts in the gut microbiota influenced host intestinal metabolism, we performed untargeted fecal metabolomics on samples taken before and after vaccination. Partial least squares discriminant analysis (PLS‐DA) revealed significant metabolic reprogramming following immunization with both CHIKV mRNA vaccines, closely paralleling microbiota divergence (Figure 4A, Figure S5A). Differential abundance analysis identified metabolites significantly enriched or depleted post‐vaccination (Figure 4B). KEGG enrichment analysis highlighted purine metabolism as the most prominently perturbed pathway (Figure 4C,D). Variable importance in projection (VIP) analysis identified xanthine and uric acid as key discriminatory metabolites in both V1 and V2 groups (Figure S5B). To delineate the dynamics of vaccine‐induced metabolic shifts, we separately profiled temporal changes in metabolite abundances before and after immunization (Figure S5C). Subsequently, UpSet analysis was performed to identify metabolites consistently upregulated post‐vaccination in both V1 and V2. Functional enrichment analysis of these persistently elevated metabolites revealed enrichment in pathways including thiamine metabolism, biosynthesis of alkaloids derived from histidine and purines, as well as purine metabolism (Figure S5D). Given the central role of purine metabolism in cellular energy homeostasis, we compared key metabolites in energy metabolic pathways across V_BG, V, CV and CHIKV‐Ctrl groups. The results showed that key metabolites within the energy metabolic flow centered on purine metabolism were consistently upregulated following vaccination. Furthermore, both V1 and V2 robustly upregulated energy‐related metabolites, particularly those within the purine pathway (Figure 4E,F). Notably, this activation was largely restricted to the vaccination phase and was not observed following viral challenge. However, xanthine and succinic acid were significantly elevated in infected animals (Figure 5A), indicating that vaccine‐induced immunity may independently lead to partial metabolic activation. To explore host–microbe–metabolite interactions, we constructed a correlation matrix of key vaccine‐perturbed microbes and metabolites. Metabolites significantly associated with *Segatella copri* were enriched in glutamate metabolism and purine metabolism (Figure S5E). Specifically, purine metabolites—including xanthine and uric acid—were significantly and positively correlated with both *Segatella copri* and *Prevotella* sp. (Figure S5F), indicating that microbes may contribute to metabolic reprogramming.

**FIGURE 4:**
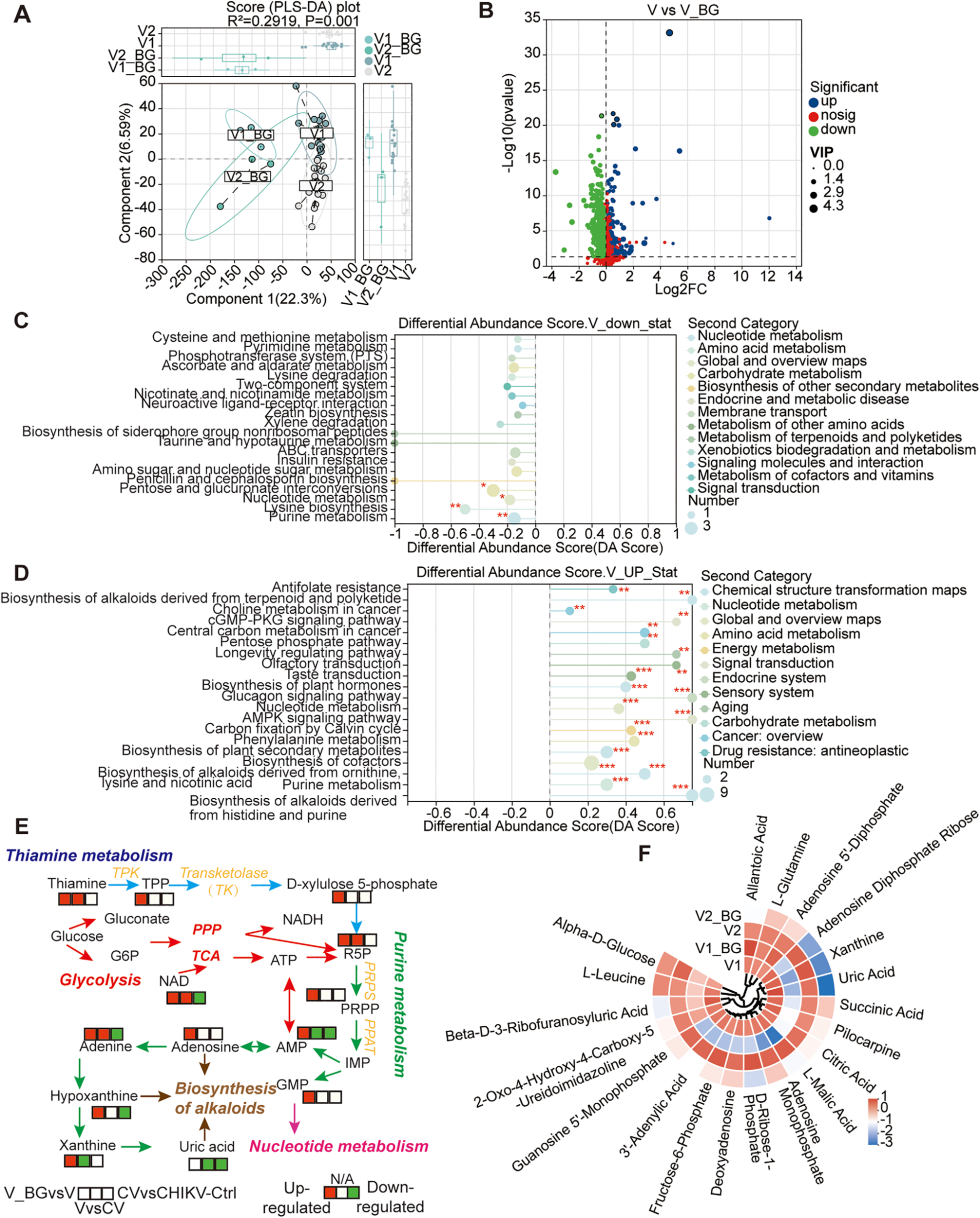
Metabolic reprogramming of the gut microenvironment after vaccination. (A) PLS‐DA of fecal metabolites before and after vaccination. Group differences were analyzed via PERMANOVA (999 permutations). *R* ^2^ was used to interpret the degree of difference among groups. The threshold for statistical significance was set at *p* < 0.05. (B) Volcano plot showing differentially abundant metabolites before and after vaccination. KEGG pathway enrichment of significantly downregulated (C) and upregulated (D) metabolites. Pathway differential abundance (DA) scores indicate the degree of enrichment. (E) Key metabolic pathways that were significantly enriched in differential metabolites, as well as their up‐ and downstream pathways with changes in the abundance of key metabolites between groups. V_BG (pre‐vaccine immunization background group), V (vaccine immunization group), CV (vaccine immunization infection group), and CHIKV‐Ctrl (unvaccinated infection group). (F) Heatmap of metabolite abundance in the intestinal purine metabolic pathway before and after vaccine immunization.

**FIGURE 5:**
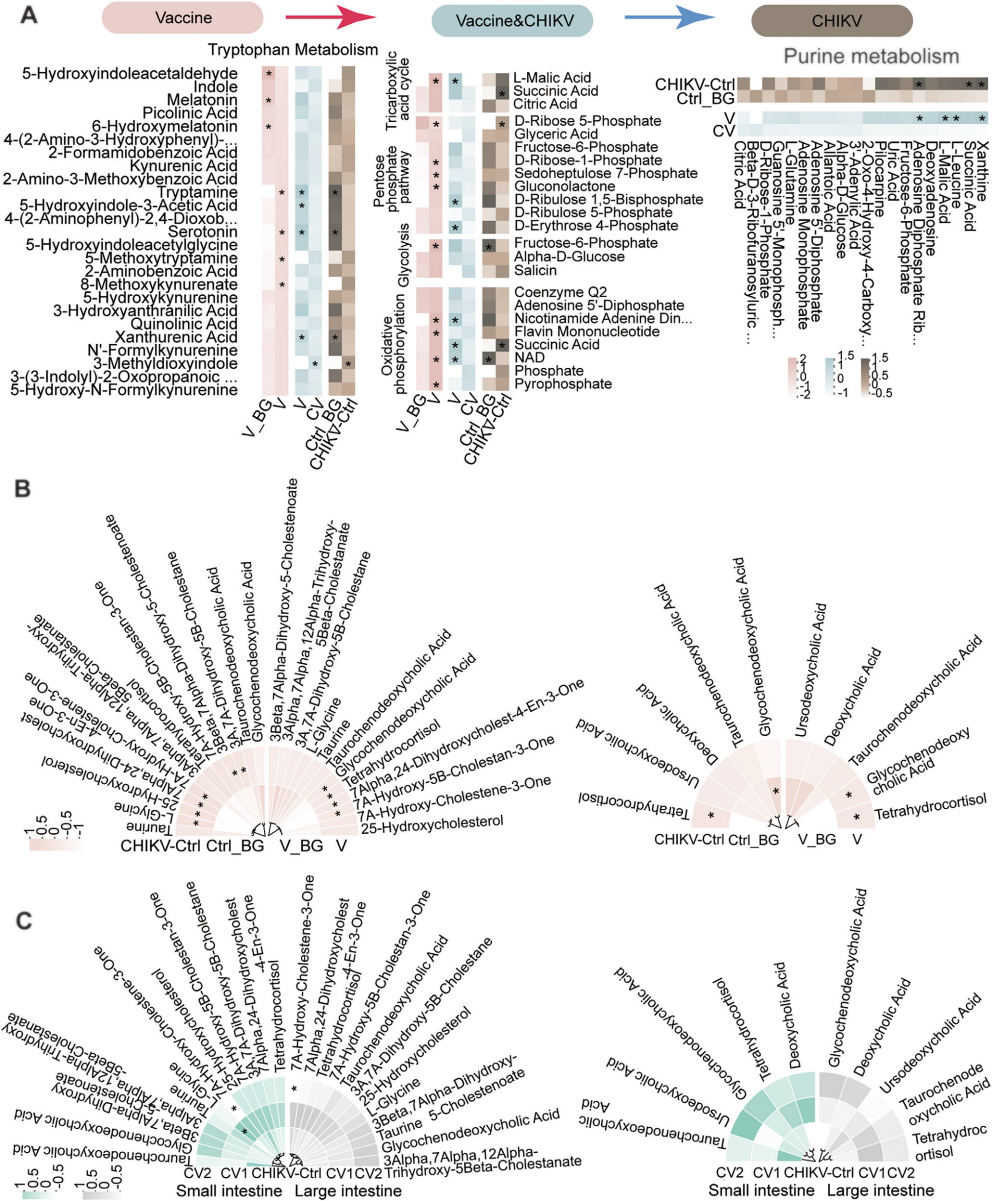
Dynamic alterations in key metabolic pathways after vaccination and infection. (A) Heatmaps showing the abundance of key metabolites related to tryptophan metabolism, energy metabolism, and purine metabolism across V_BG (pre‐vaccine immunization background group), V (vaccine immunization group), CV (vaccine immunization infection group), and CHIKV_Ctrl (unvaccinated infection group). Paired comparisons between two time points within the same animals were performed using the Wilcoxon signed‐rank test with FDR correction. The threshold for statistical significance was set at (*q < 0.05). (B) Heatmap of bile acid‐related metabolites in the infection background (C_BG), V_BG, V, and C groups. (C) Heatmap of bile acid‐related metabolites in the small and large intestinal contents of the CV and C groups. Statistical analysis via Kruskal–Wallis test (Dunn's post hoc) with FDR correction (*q < 0.05).

Given that bile acid dysregulation is a marker of CHIKV infection [16], we first characterized fecal bile acid profiles to assess the impact of vaccination on bile acid metabolism. The results confirmed that CHIKV infection induces substantial alterations in intestinal bile acid metabolism. Compared with the baseline control group (Ctrl_BG), CHIKV infection induced marked alterations in bile acid‐related metabolites, with several secondary bile acid‐ and sterol‐related metabolites—including 25‐hydroxycholesterol, 7α,24‐dihydroxycholest‐4‐en‐3‐one, 7α‐hydroxy‐5β‐cholestan‐3‐one, and tetrahydrocortisol—showing significantly increased abundance. Notably, a similar shift was also observed following vaccination alone, indicating that CHIKV mRNA immunization can induce bile acid metabolic changes that partially recapitulate those associated with direct infection (Figure 5B). We next characterized bile acid–related metabolites in the small‐ and large‐intestinal contents to assess how prior vaccination influenced bile acid metabolism following CHIKV challenge, relative to direct infection. Untargeted metabolomics revealed no significant differences in most bile acid‐related metabolites between the vaccinated‐challenged and CHIKV‐Ctrl groups, except for 7α‐hydroxycholestene‐3‐one and L‐glycine, which were significantly decreased and increased in CV2 and CV1, respectively (Figure 5C).

Collectively, both V1 and V2 induced broadly concordant intestinal metabolic remodeling before viral challenge, characterized by prominent activation of purine metabolism and sustained increases in xanthine and uric acid. These metabolic changes were closely associated with vaccination‐induced microbial shifts, particularly the enrichment of *Segatella copri* and *Prevotella* spp. Vaccination also altered bile acid‐related metabolites in a pattern partially resembling that observed during CHIKV infection. Thus, despite their distinct immunogenicity and protective efficacy, the two vaccines elicited shared microbial‐associated metabolic responses involving purine and bile acid pathways.

### 2.4 Vaccination Induces a Protective Intestinal Immune Microenvironment and Promotes Epithelial Barrier Remodeling in the Intestinal Mucosa

To evaluate how CHIKV vaccination modulated host intestinal immunity and epithelial changes in response to infection, we performed proteomic analyses of key intestinal tissues, including the small intestine, colon, and Peyer's patches, from animals in the vaccinated‐challenged (CV) and CHIKV‐Ctrl groups. As a major immunological site, the small intestine exhibited substantial changes in the CV group, with 935 and 191 proteins significantly up‐ and downregulated compared with CHIKV‐Ctrl, respectively (Figure 6A). Functional enrichment analysis revealed upregulation of pathways associated with viral infection and immunological processes (Figure 6B). These notably included proteins involved in primary and secondary immune responses, such as the Fc epsilon RI signaling pathway, Fc gamma R‐mediated phagocytosis, and the B cell receptor signaling pathway (Figure 6C), suggesting that vaccination enhanced antibody‐mediated phagocytic and regulatory functions. Gene set enrichment analysis (GSEA) further confirmed the activation of these immune‐related pathways in the CV group (Figure 6D). Proteins involved in the primary immune response, intestinal B cell responses, and IgA‐related pathways were significantly upregulated in the vaccinated animals following CHIKV challenge compared with CHIKV‐Ctrl (Figure 6E). Notably, key proteins such as CD8α, RFXAP (a transcriptional activator of MHC II), and IGHG1/IGHG3 (IgG heavy chains) were markedly upregulated in the CV group (Figure 6F). Comparative proteomic profiling of the small and large intestines further confirmed increased expression of proteins involved in antigen presentation, pathogen clearance, and T/B cell responses in the vaccinated group (Figure 7A), although interferon‐induced proteins were decreased compared with CHIKV‐Ctrl. Given the central immunological roles of Peyer's patches and mesenteric lymph nodes (MLNs), we also performed proteomic profiling on these tissues. GSEA showed significant enrichment of pathways related to antigen processing and presentation, T‐cell receptor signaling, and the intestinal immune network for IgA production in the CV group (Figure 7B). Specifically, proteins related to antigen presentation and clearance were highly expressed in Peyer's patches. By contrast, MLNs from the CHIKV‐Ctrl group exhibited greater enrichment of inflammatory mediators, interferon‐induced proteins, and T‐cell‐related pathways (Figure 7C). Consistent with these findings, further analyses of immunoregulatory and inflammatory cytokines confirmed higher expression of pro‐inflammatory cytokines—including MIP1A, MIP1B, and VEGFA—in MLNs from the CHIKV‐Ctrl group. Conversely, higher levels of immunoregulatory cytokines, such as IL‐15, were observed in the small intestines of the CV1 and CV2 animals (Figure S6). Immunofluorescence staining also confirmed that the abundance of CD4^+^ T cells was increased in intestinal lymphoid tissues of infected animals (Figure S7A). Finally, we further confirmed the protective effect of the vaccines on the intestinal barrier after infection by measuring the protein levels of key epithelial marker molecules. The results showed that the small and large intestinal tissues of the vaccine‐immunized infected group exhibited higher levels of tight junction and mucosal barrier proteins. Epithelial repair and cell homeostasis‐associated proteins were also increased, especially in the small intestine (Figure 7D). Alcian blue and periodic acid‐Schiff (AB‐PAS) staining of mucosal goblet cells post‐CHIKV infection further validated vaccine‐mediated protection of the intestinal mucosal barrier (Figure S7B).

**FIGURE 6:**
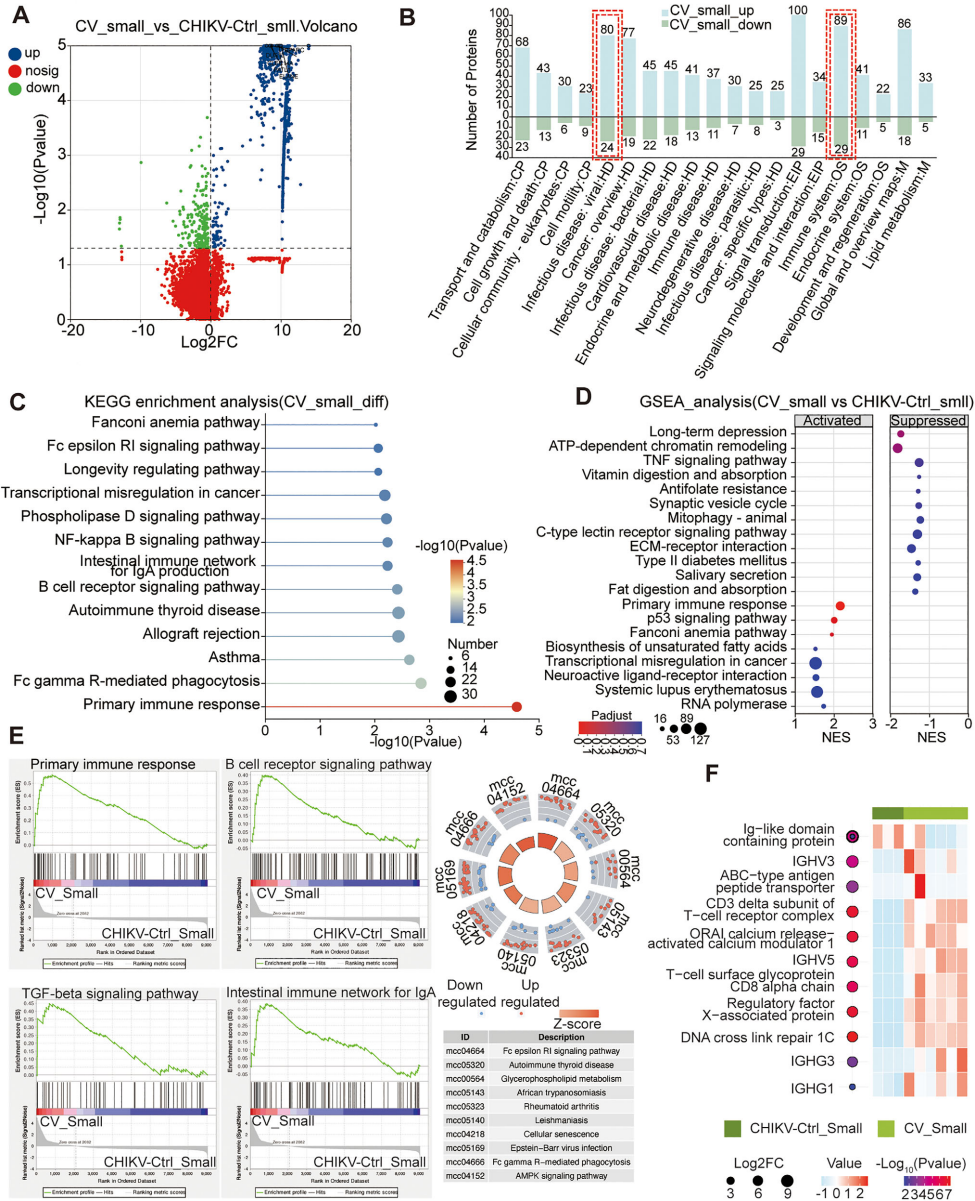
Gut proteomics revealed the antiviral mechanisms post‐vaccination. As shown in Figure 1A, samples of intestinal tissues were collected at the designated time points for proteomic analysis. Raw data were processed as described in the “Methods” section. (A) Volcano plot of differentially expressed proteins (DEPs) in the small intestine between the CV and CHIKV‐Ctrl groups. (B) KEGG enrichment analysis of significantly up‐ and downregulated proteins (CV vs. CHIKV‐Ctrl). The red box shows the function with the highest enrichment among KEGG human diseases (HD) and organismal systems (OS) functions (Level 2), and the corresponding secondary functions (C). (D) Gene set enrichment analysis (GSEA) identified activated (NES > 0) and suppressed (NES < 0) biological functions in CV versus CHIKV‐Ctrl. (E) GSEA‐enriched functions and circular plot of the corresponding DEPs, showing that most proteins were upregulated in CV. (F) Heatmap showing key proteins upregulated in the CV group (small intestine) within the enriched functions.

**FIGURE 7:**
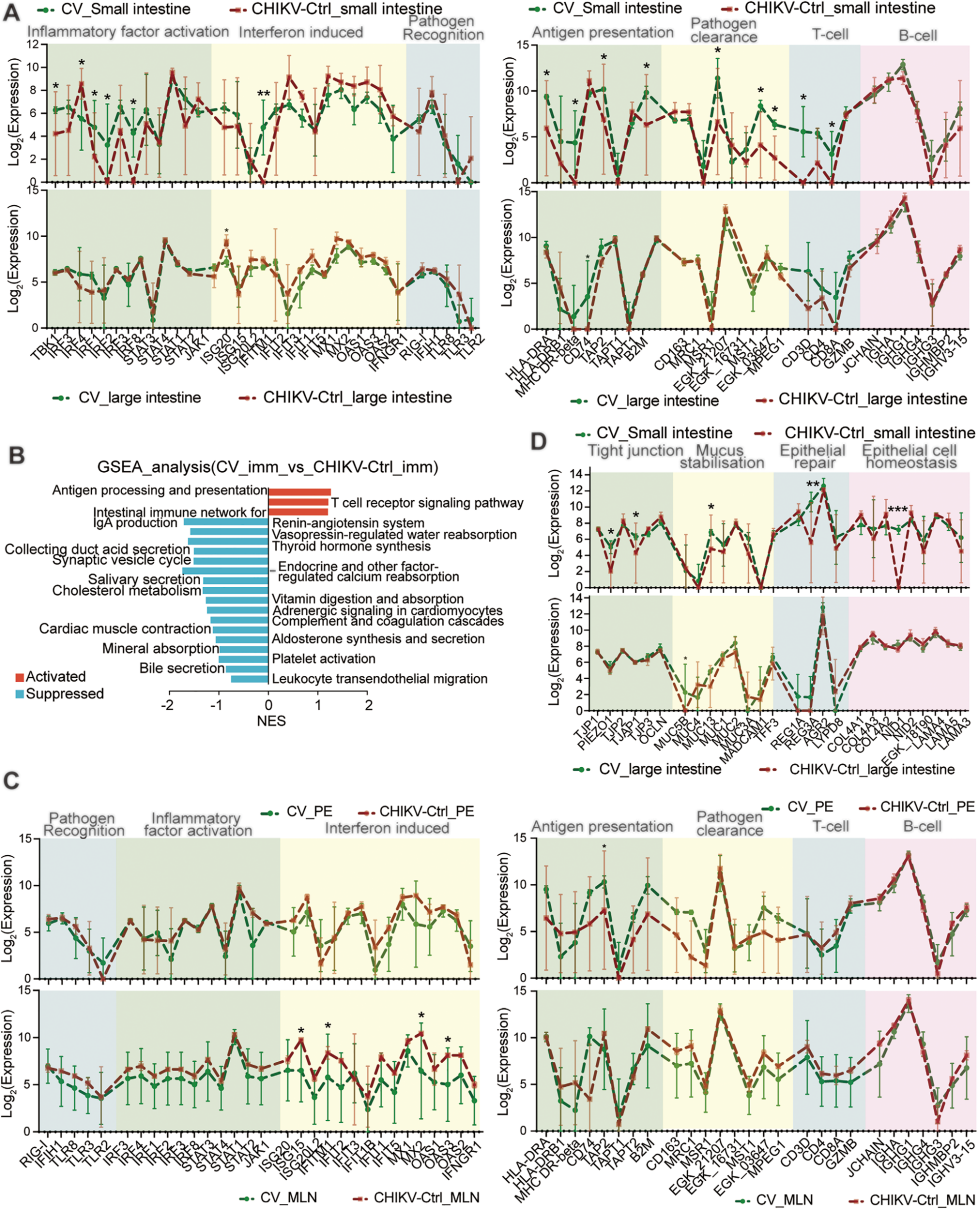
Expression characteristics of key proteins involved in immunoregulation and antiviral responses in intestinal tissues of vaccinated animals. (A) Expression of critical immune and antiviral proteins in CV (vaccine immunization infection group) versus CHIKV_Ctrl (unvaccinated infection group). Statistical analysis via Wilcoxon rank‐sum test, FDR corrected (*q < 0.05). (B) GSEA of differentially expressed proteins in Peyer's patches and mesenteric lymph nodes (MLNs) between the CV and CHIKV‐Ctrl groups. (C) Comparative expression of immune‐related proteins in Peyer's patches and MLNs between the CV and CHIKV‐Ctrl groups. Statistical analysis via Wilcoxon rank‐sum test, FDR corrected (*q < 0.05). (D) Expression of tight junction and epithelial barrier proteins in intestinal tissues (left: small intestine; right: large intestine) between the CV and CHIKV‐Ctrl. Statistical tests: Wilcoxon rank‐sum test, FDR corrected (*q < 0.05).

Overall, CHIKV vaccination enhanced the activation of antigen presentation, B cell responses, and IgA‐associated pathways in the small intestine, with elevated expression of key proteins such as CD8α, RFXAP, and IGHG1 following infection. Compared with direct infection, the vaccinated group exhibited stronger immune activation in Peyer's patches, whereas MLNs in the direct infection group showed higher levels of pro‐inflammatory and interferon‐induced factors. Proteins associated with the intestinal epithelial barrier and repair were more highly expressed in vaccinated animals, particularly in the small intestine.

## 3 Discussion

The interplay between vaccine‐induced immunity and the intestinal microenvironment is emerging as a critical determinant of systemic immune homeostasis and vaccine efficacy [17]. There is evidence that vaccine immunization can reshape gut microbial composition and function, with potential consequences for host metabolism and mucosal immunity [18]. However, due to the inherent complexity and variability of the human microbiome, our current understanding of how vaccines modulate the gut ecosystem remains limited [19]. In particular, there is a striking absence of studies directly comparing the effects of vaccination and natural viral infection on gut microbial and immune dynamics within the same experimental framework. Such comparative insights are essential for elucidating how CHIKV mRNA vaccination, in the absence of replicating pathogens, remodels gut ecology and shapes downstream mucosal immune responses. Addressing this gap, our study leveraged two previously validated CHIKV mRNA vaccines in a NHP model and integrated metagenomics, metabolomics, and proteomics to systematically characterize the intestinal microbiota, metabolic alterations, and gut immune characteristics following vaccination and subsequent viral challenge.

The effects of the gut microbiota on vaccine efficacy have been repeatedly demonstrated. Swaminathan et al. showed that antibiotic depletion of the gut microbiota significantly impaired vaccine‐induced immune responses, as evidenced by reductions in antigen‐specific IgM, IgG, and virus‐neutralizing antibody titers, as well as decreases in cellular immune responses (lymph node T follicular helper cells, germinal center B cells, and plasma cells). Restoring the gut microbiota before vaccination rescued these impaired antibody responses [20]. Similarly, Hagan et al. reported that antibiotic‐induced disruption of gut microbial communities impaired IgG1 responses to the trivalent inactivated influenza vaccine (TIV) [21]. More recently, Feng et al. provided direct evidence in humans that antibiotic treatment substantially weakens humoral immune responses to rabies vaccination, reducing rabies‐specific IgG, with impaired plasmablast and B cell clonal expansion [14]. Several mechanisms have been proposed to explain how the gut microbiota modulate host responses to vaccination. These include microbial‐derived cross‐reactive epitopes shared with vaccine antigens [22], immunomodulatory microbial metabolites such as short‐chain fatty acids (SCFAs), tryptophan catabolites, and bile acids [21, 23], as well as microbial pathogen‐associated molecular patterns (PAMPs) or damage‐associated molecular patterns (DAMPs), which stimulate pattern recognition receptors (PRRs) and may serve as natural adjuvants [24]. Together, these factors shape vaccine immunogenicity and modulate the activity of key immune‐cell subsets [25]. By contrast, the effects of vaccination on the gut environment remain poorly understood.

In this study, we provide direct evidence that CHIKV mRNA vaccination, even in the absence of viral replication, is sufficient to reshape the host gut microenvironment. Notably, vaccination was associated with gut microbial alterations toward a state resembling that of active infection. Longitudinal metagenomic analyses revealed that even a single dose of mRNA vaccination was sufficient to perturb gut microbial composition and diversity. These vaccine‐induced microbial shifts paralleled those observed during CHIKV infection, including depletion of Bacillota and expansion of Bacteroidota and Spirochaetota. At the genus and species level, enrichment of *Segatella copri* and *Treponema* spp., coupled with depletion of beneficial *Lactobacillus* spp., was shared across vaccinated and infected groups [5]. *Prevotella* spp. are not considered strict pathogens, but their enrichment has been associated with various immunological and inflammatory disorders [26]. In particular, the proinflammatory role of *Segatella copri* in musculoskeletal inflammation—such as RA and ankylosing spondylitis—has been well documented [27, 28, 29]. Maeda et al. reported that the abundance of *Segatella copri* was significantly increased in the feces of RA patients, and that fecal microbiota transplantation into mice induced RA‐like immune responses and phenotypes [30]. Mechanistically, high levels of *Prevotella* are thought to activate intestinal immune cell and chemokine responses, particularly Th17 and IFN‐γ responses [26]. IL‐17, mainly secreted by Th17 cells, induces epithelial cells to produce proinflammatory cytokines and chemokines, sustaining inflammation and promoting the activation of fibroblast‐like synoviocytes and osteoclasts, which contribute to joint inflammation and bone destruction in arthritis [31, 32]. Consistently, a previous study implicated IL‐17 in the pathogenesis of chronic arthralgia following CHIKV infection. In rhesus macaque models, CHIKV infection induced high IL‐17 expression in the gut, prompting the concept of a CHIKV‐driven “gut‐joint axis” [32]. Furthermore, our previous work demonstrated that consistent shifts in gut microbiota composition—especially *Segatella copri*—have been repeatedly observed across animal models of CHIKV infection [5, 33]. Even under microbiota depletion, CHIKV infection robustly increased the abundance of this species, establishing a *Segatella copri*‐dominated enterotype. The results of this study show that both V1 and V2 immunizations induced increases in *Segatella copri* abundance and IL‐17 expression.

We also observed that vaccination induced significant metabolic reprogramming, particularly enhancing purine metabolism and increasing the levels of key metabolites including xanthine and uric acid—shifts strongly correlated with altered microbial composition. Intestinal inflammation is a known driver of purine metabolic disturbances. Wu et al. reported dysregulated purine metabolism in ulcerative colitis (UC), and showed that rhein treatment ameliorated inflammation and normalized purine pathway activity [34]. Duan et al. demonstrated that endoplasmic reticulum (ER) stress in intestinal epithelial cells (IECs) upregulated Duoxa2/Duox2 expression, increasing reactive oxygen species (ROS) production and activating the purine metabolic pathway, thereby promoting xanthine production and enhancing Th17 cell differentiation. Conversely, colonization with Th17‐inducing microbes exacerbated ER stress and further increased purine metabolites in IECs [35]. Bile acid dysregulation is a representative feature during CHIKV infection: Previous studies have shown that CHIKV infection is accompanied by substantial remodeling of primary, secondary, and conjugated bile acid profiles in both rhesus macaques [33] and mice. Winkler et al. demonstrated that intestinal bile acid‐transforming bacteria, particularly *Clostridium scindens* with 7α‐dehydroxylation activity, generate secondary bile acids such as deoxycholic acid (DCA), which enhance TLR7–MyD88‐dependent type I interferon responses in plasmacytoid dendritic cells following CHIKV infection to maintain an antiviral state in circulating monocytes and thereby limit viremia, systemic dissemination, and onward transmission to mosquitoes [16]. In the present study, CHIKV mRNA vaccination alone was sufficient to induce shifts in primary and secondary bile acid‐related metabolites, with an overall trend resembling that observed during active infection. These findings suggest that the convergence of the gut microenvironment induced by CHIKV vaccination with that of direct infection is reflected not only in the microbial composition but also in the remodeling of the gut metabolic landscape. Accordingly, we hypothesized that the antigenic profiles presented by the mRNA vaccines used in this study—whether encoding the full‐length CHIKV structural proteins (E1, E2, E3, and C) or the key envelope proteins (E1, E2, and E3)—may elicit an infection‐like gut ecological state [35]: reduced microbial diversity, depletion of selected *Lactobacillus* species, enrichment of *Segatella copri* and *Treponema* spp., and alterations in purine‐ and bile acid‐related metabolism. Potential mechanisms include the possibility that CHIKV mRNA vaccination may similarly drive *Segatella copri* enrichment and IL‐17 expression—factors that may contribute to the observed reprogramming of purine and bile acid metabolism. Moreover, given the central role of purine metabolism in energy balance, the transient metabolic pressure induced by vaccine‐triggered antigen expression may represent an additional mechanism underlying the observed purine pathway activation [36, 37] (Figure S8).

In addition to modulating microbial and metabolic dynamics, CHIKV vaccination resulted in distinctive immune activation in the gut following viral challenge, with an enhanced immune response and improved intestinal barrier integrity, characterized by elevated expression of proteins related to antigen presentation, B‐cell activation, and antibody production—including proteins associated with intestinal immunoglobulin production—as well as tight junction and epithelial repair markers. Proteomic analysis of small intestinal tissues and associated lymphoid structures revealed upregulation of canonical adaptive immune regulators, including CD8α, RFXAP, and IgG heavy chain variants. These molecules not only serve as markers of effective systemic immune priming, but are also increasingly being recognized for their roles in shaping local intestinal immune responses [38], particularly within Peyer's patches where antigen sampling and class switching to IgA occur [39]. The enhanced expression of intestinal IgA‐associated proteins post‐vaccination aligns with the concept of “mucosal imprinting” by systemic immunization, an area of growing interest in vaccinology [40, 41, 42]. However, the directly infected group exhibited more pronounced interferon‐driven signatures and increased inflammatory proteins in GI tissues—particularly within MLNs and Peyer's patches—the vaccinated group maintained a more regulated response. This included preserved antigen processing capacity, moderate inflammatory activation, and elevated expression of epithelial tight junction proteins. This divergence suggests that vaccination preconditioned the GI immune landscape to mount a coordinated and less tissue‐damaging response, likely by reprogramming innate‐adaptive crosstalk at inductive sites. Notably, the expression of IL‐15, a cytokine involved in intraepithelial lymphocyte survival and epithelial barrier regulation [43], was higher in the vaccinated group, suggesting a role for GI cytokine homeostasis in protective immunity [44].

Taken together, these results suggest that CHIKV mRNA vaccination establishes a primed intestinal immune landscape capable of mounting a rapid and adaptive response to viral exposure, while preserving tissue architecture and barrier function. This highlights the ability of systemic vaccines to exert local effects in the GI tract, even against non‐enteric viruses. Overall, these findings suggest that CHIKV mRNA vaccination induces a coordinated intestinal response involving microbial, metabolic, and cytokine remodeling that partially overlaps with the response to active infection. However, infection‐like ecological remodeling and infection‐associated pathology should not be regarded as equivalent biological processes. Because both vaccines express overlapping CHIKV structural antigens and elicited shared microbial, metabolic, and cytokine alterations, one possible explanation is that systemic immune responses following vaccination engage host–gut signaling pathways that partially overlap with those involved in infection; these similarities may reflect CHIKV antigen‐specific effects [45]. Unlike active infection, vaccination occurs in the absence of sustained viral replication and establishes adaptive immune memory before challenge. This immune priming may enable rapid viral control and limit tissue‐damaging inflammatory responses, even though selected microbiota and metabolic signatures resemble those observed during infection. Accordingly, the enrichment of *Segatella copri* and depletion of selected *Lactobacillus* species should not be interpreted as direct evidence of either protection or pathological dysbiosis. Rather, these changes may represent components of a broader vaccination‐associated intestinal ecological response (Figure S8). Our study provides multi‐omics evidence that parenteral mRNA vaccination against CHIKV, even in the absence of viral infection, can profoundly remodel the host intestinal ecosystem—recapitulating key microbial, metabolic, and GI immune features typically consistent with active CHIKV infection. However, several limitations remain. First, the precise mechanisms underlying CHIKV mRNA vaccination‐associated remodeling of the gut microenvironment require further investigation. Without an empty‐LNP control or an irrelevant‐antigen mRNA control, the relative contributions of CHIKV antigen expression, the mRNA platform, and lipid nanoparticle delivery remain to be further clarified. Second, ethical constraints on animal numbers prevented us from systematically resolving the spatiotemporal dynamics of intestinal immune responses post‐vaccination. Third, we did not directly measure mucosal immunoglobulins such as sIgA, limiting our ability to fully characterize antigen‐specific responses at the gut interface. Future work employing single‐cell profiling, spatial transcriptomics, and direct quantification of mucosal antibodies will be essential to further delineate the mechanisms linking systemic immunization to intestinal immunomodulation. Our findings underscore the importance of considering intestinal immune responses and host‐microbiota interactions as integral components of vaccine‐elicited protection, especially for viruses capable of systemic dissemination. These insights may inform future vaccine development and broaden our understanding of vaccine‐host interactions beyond classical systemic paradigms.

## 4 Materials and Methods

### 4.1 Animal Ethics and Biosafety

All procedures involving live chikungunya virus (CHIKV) and animal infection were performed in a biosafety level 3 (BSL‐3) facility, in accordance with its classification as a highly pathogenic agent. Experimental procedures were reviewed and approved by the Institutional Animal Care and Use Committee of the Institute of Medical Biology, Chinese Academy of Medical Sciences (IACUC approval no. DWSP20240309).

### 4.2 mRNA Vaccination of NHPs

Two in‐house mRNA vaccines, V1 and V2, encode CHIKV E1, E2, E3, and C proteins and E1, E2, and E3 proteins, respectively. Vaccine preparation, dosing regimens, and efficacy were described previously [15]. Nine male, 4‐year‐old rhesus macaques (*Macaca mulatta*) were randomly assigned to a phosphate‐buffered saline‐treated control group (Ctrl) or either vaccine group (V1 or V2). Baseline (background) samples collected before immunization from the V1 and V2 groups were designated V1BG and V2BG, respectively. Animals were individually housed under a 12‐h light/dark cycle and fed standardized commercial monkey chow, with water available ad libitum. Before vaccination, all animals were screened for pre‐existing CHIKV infection and clinically assessed to exclude underlying conditions, including arthritis and GI disorders. Fresh fecal and serum samples were collected longitudinally before and after immunization for microbiota and circulating cytokine analyses (Figure 1A). Group definitions, animal numbers, sample types, omics datasets, and comparisons are detailed in Table S1.

### 4.3 CHIKV Strains and Animal Experimental Procedures

Animals were inoculated intramuscularly in the hind limb with 2 × 10^7^ plaque‐forming units (PFU) of an Asian‐lineage CHIKV strain (GenBank: MH670649.1). Fresh fecal and serum samples were collected every 2 days during the 7‐day post‐infection period. At 7 days post‐infection (dpi), all animals were anesthetized and euthanized for GI tissue and content collection. Small‐ and large‐intestinal contents were divided into three portions for microbial metagenomics, untargeted metabolomics, and viral load quantification. Tissues collected for histology, immunofluorescence, and proteomics included the stomach, duodenum, ileum, cecum, ascending, transverse, and descending colon, rectum, Peyer's patches, and MLNs. The workflow is shown in Figure 1A. Post‐challenge samples from V1‐ and V2‐vaccinated animals were designated CV1 and CV2, respectively, and the infected negative‐control group was designated CHIKV‐Ctrl.

### 4.4 Determination of Viral Load in the Intestinal Content and GI Tissues

Viral RNA was extracted from tissue, blood, and intestinal contents using the Direct‐zol RNA Miniprep Kit (Zymo Research, Cat. No. R2052) according to the manufacturer's protocol. CHIKV genomic RNA was quantified by E1‐targeted quantitative reverse‐transcription PCR (qRT‐PCR) using standard curves to calculate copy numbers in each sample type. Primers and probe were CHIKV E1F (CTCATACCGCATCCGCATCAG), E1R (ACATTGGCCCCACAATGAATTTG), and CHIKV E1 Probe (FAM‐TCCTTAACTGTGACGGCATGGTCGCC‐BHQ1).

### 4.5 Histopathological Analysis

GI tissues were fixed in 10% neutral‐buffered formalin at 25°C for at least 10 days after collection, paraffin‐embedded, sectioned, and stained with hematoxylin and eosin (H&E). Slides were digitized using the 3D HISTECH system (Hungary). Experienced pathologists assessed each section under single‐blind conditions for epithelial cell integrity, tissue edema, hemorrhage or ulceration, and inflammatory cell infiltration using a semiquantitative scale from 0 (absent) to 4 (severe). Histopathological scores were compiled for statistical analysis.

### 4.6 Immunofluorescence Analysis

Paraffin‐embedded tissue sections (3 µm) were incubated with primary antibodies against ZO‐1 (ThermoFisher, Cat. No. 33‐9100; 1:100), EpCAM (Abcam, Cat. No. ab282457; 1:100), and CD4 (Abcam, Cat. No. ab133616; 1:5000), followed by corresponding fluorescence‐conjugated secondary antibodies. Nuclei were counterstained with DAPI, and slides were scanned using the 3D HISTECH imaging system. Marker‐positive cells were quantified using ImageJ, with the staining index defined as their percentage among all cells per section. Data were visualized using GraphPad Prism (v8.0).

### 4.7 AB‐PAS Staining

Paraffin‐embedded GI tissues were sectioned at 3 µm, stained with AB‐PAS (Solarbio, Cat. No. G1049) according to the manufacturer's protocol, and digitized using the 3D HISTECH scanning system. Goblet cells were quantified as previously described. Each slide was divided into four quadrants, with five crypts randomly selected per quadrant. Only goblet cells ≥ 150 µm beneath the surface epithelium in longitudinally cut crypts were counted. Between‐group differences were assessed using Welch's *t*‐test, and data were visualized using GraphPad Prism (v8.0).

### 4.8 Compositional and Functional Profiling of Intestinal Contents via Metagenomic Sequencing

Feces were collected directly into DNA/RNA Shield Lysis Tubes (ZymoBIOMICS, R1103‐50) for viral inactivation. Microbial DNA was extracted using the ZymoBIOMICS DNA Miniprep Kit (D4300) following the manufacturer's instructions. DNA integrity and purity were assessed by 1% agarose gel electrophoresis, and DNA was quantified using a NanoDrop ND2000 (Thermo Scientific, USA). DNA was fragmented to ∼400 bp on average for library preparation using the NEXTFLEX Rapid DNA‐Seq Kit (BioScientific, Austin, TX, USA). Majorbio Bio‐Pharm Technology Co., Ltd. (Shanghai, China) performed paired‐end sequencing on an Illumina NovaSeq 6000 with the S4 Reagent Kit. FASTQ tools removed adapters, low‐quality reads (length < 50 bp, quality score < 20), and reads with ambiguous bases. BWA (v0.7.17; http://bio‐bwa.sourceforge.net) removed host‐derived reads. High‐quality reads were assembled using Multiple_MEGAHIT (v1.1.2; https://github.com/voutcn/megahit), retaining contigs > 300 bp. Open reading frames (ORFs) were predicted using Prodigal and clustered into a non‐redundant gene catalog using CD‐HIT (v4.7; http://weizhongli‐lab.org/cd‐hit/) at 90% sequence identity and 90% coverage. For quantification, high‐quality reads from each sample were mapped to the catalog using SOAPaligner (v2.21; https://github.com/ShujiaHuang/SOAPaligner) at a 95% identity cutoff. Genes were taxonomically annotated against NCBI NR using DIAMOND (v2.0.13; *e*‐value < 1 × 10^−^^5^, best‐hit mode) and functionally annotated against COG, KEGG, VFDB, and CAZy.

### 4.9 Non‐Targeted Metabolomic Profiling of Intestinal Contents by LC–MS

Intestinal content (∼100 mg) was homogenized in 2‐mL centrifuge tubes with 6‐mm steel beads for 6 min at −10°C and 50 Hz. Metabolites were extracted with 80% methanol (methanol:water, 4:1, v/v) containing 0.02 mg/mL L‐2‐chlorophenylalanine as the internal standard. Extracts were analyzed using a Thermo Scientific UHPLC‐Q Exactive HF‐X system with an ACQUITY HSS T3 column (100 mm × 2.1 mm, 1.8 µm; Waters, USA). Mobile phases A and B were water:acetonitrile (95:5, v/v) and acetonitrile:isopropanol:water (47.5:47.5:5, v/v/v), respectively, both containing 0.1% formic acid. Flow rate, column temperature, and injection volume were 0.40 mL/min, 40°C, and 3 µL, respectively. Positive and negative electrospray ionization settings were as follows: source temperature, 425°C; sheath/auxiliary gas flow, 50/13 arbitrary units; spray voltage, ±3500 V; and stepped normalized collision energy (NCE), 20–40–60 V for MS/MS. Full MS and MS2 resolutions were 60,000 and 7500, respectively. Data‐dependent acquisition (DDA) covered *m*/*z* 70–1050. Progenesis QI (Waters Corporation, Milford, USA) generated a three‐dimensional matrix from raw LC–MS data, excluding internal standard signals and known artifacts (noise, column bleed, and reagent peaks). Metabolites were annotated by matching MS features against HMDB (http://www.hmdb.ca/). Features detected in ≥ 80% of samples within any group were retained. Missing values were imputed with the minimum detected concentration, and intensities were normalized to each sample's total ion signal (sum normalization). Variables with relative standard deviation (RSD) > 30% in QC samples were excluded, followed by log10 transformation for statistical analysis.

### 4.10 Multiplex Quantification of Inflammatory Cytokines

Cytokines and related mediators in blood and tissues were quantified using the MILLIPLEX MAP Non‐Human Primate Cytokine Magnetic Bead Panel (Millipore, Cat. No. PRCYT2MAG40K) on a Bio‐Plex multiplex system (Bio‐Rad) following the manufacturer's protocol. Analytes included interleukin (IL)‐1α, IL‐1β, IL‐2, IL‐4, IL‐6, IL‐8, IL‐10, IL‐12/23(p40), IL‐15, IL‐17A, IL‐18, granulocyte colony‐stimulating factor (G‐CSF), granulocyte‐macrophage colony‐stimulating factor (GM‐CSF), monocyte chemoattractant protein‐1 (MCP‐1/CCL2), macrophage inflammatory proteins MIP‐1α (CCL3) and MIP‐1β (CCL4), transforming growth factor‐alpha (TGF‐α), vascular endothelial growth factor (VEGF), and CD40L.

### 4.11 Gut Tissue Proteomic Profiling and Bioinformatic Analysis

Tissues were kept on ice and homogenized in 8 M urea and 1% SDS containing an EDTA‐free protease inhibitor cocktail (cOmplete, Mini, Roche) using a SCIENTZ‐48L tissue grinder (SCIENTZ, China; three 180‐s cycles), followed by 30‐min non‐contact cryogenic ultrasonication. Lysates were centrifuged at 16,000 × *g* for 30 min at 8°C, and supernatant protein concentrations were measured using a bicinchoninic acid assay kit (Thermo Scientific) following the manufacturer's instructions.

For each sample, 100 µg protein was reconstituted in 100 mM triethylammonium bicarbonate (TEAB), reduced with 10 mM tris(2‐carboxyethyl)phosphine for 60 min at 37°C, and alkylated with 40 mM iodoacetamide for 40 min at room temperature in darkness. After centrifugation at 10,000 × *g* for 20 min at 4°C, pellets were resuspended in 100 µL of 100 mM TEAB. Majorbio performed the subsequent proteomic analysis. Proteins were digested overnight at 37°C with sequencing‐grade trypsin (enzyme, 1:50, w/w). Peptides were vacuum‐dried, reconstituted in 0.1% trifluoroacetic acid, desalted using Oasis HLB cartridges (Waters), and vacuum‐dried again. Concentrations were estimated from aromatic amino acid absorbance at 280 nm using a NanoDrop One UV–Vis spectrophotometer (Thermo Scientific).

Equal amounts of desalted peptides were analyzed by liquid chromatography–mass spectrometry using a Vanquish Neo UHPLC coupled to a timsTOF HT (Bruker, Germany) through a Compass HyStar interface. Separation used a custom‐packed column (15 cm × 100 µm, 1.7 µm particles) and an 8‐min gradient with aqueous solvents containing 0.1% formic acid and either 2% (A) or 80% (B) acetonitrile. Data‐independent acquisition (DIA) used parallel accumulation–serial fragmentation, with MS1 and MS2 ranges of *m*/*z* 70–1050 and 150–2000, respectively. Raw DIA files were processed using Spectronaut v19 (Biognosys), selecting up to six peptides per protein and three fragment ions per peptide for quantification. Peptide and protein identifications required a false discovery rate ≤ 1%, with peptide confidence scores ≥ 99%. Extracted‐ion chromatogram mass tolerance was ≤ 75 ppm; shared peptides and those containing post‐translational modifications were excluded from quantification.

Proteins were identified against the UniProt *Macaca mulatta* reference proteome (https://www.uniprot.org/taxonomy/9544), requiring ≥ 1 unique peptide for confident identification. Abundance was calculated by summing unique‐peptide peak areas. Analyses used R v3.5.1, with differential expression defined by |log2FC| > 1 and *p* < 0.05. Principal‐coordinate analysis was performed, and between‐group differences were tested using PERMANOVA (999 permutations; vegan). Correlation heatmaps used pheatmap, and differentially expressed proteins underwent Gene Ontology and Kyoto Encyclopedia of Genes and Genomes enrichment analyses.

## Author Contributions

S.L., Y.W., and H.C. designed the study. H.C., W.X., and S.L. wrote the manuscript. H.C., Yun Y., C.T., W.X., X.L., Yuxia Y., J.W., Y.Z., H.Y., Q.H., W.Q., W.Y., H.W., and D.W. performed experiments. H.C., Yun Y., and C.T. analyzed data. All authors have read, edited, and approved the final manuscript.

## Funding

This work was supported by grants from the Prevention and Control of Emerging and Major Infectious Diseases‐National Science and Technology Major Project (2025ZD01900700), Yunnan Major science and Technology Special Project Program (202602AS100009), Yunnan Science and Technology Talent and Platform Program (202605AS350013,202505AK340009), CAMS Innovation Fund for Medical Sciences (2022‐I2M‐3‐001), Science and Technology Leading Talent Program of Yunnan Province (202405AB350002), High‐Level Medical Talent Training Program of Yunnan Province (L‐2024018), Fundamental Research Funds for the Central Universities (3332022072), and Key Project of Basic Research in Yunnan Province (202401AS070049).

## Ethics Statement

All animal experiments in this study were approved by the Institutional Animal Care and Use Committee of the Institute of Medical Biology, Chinese Academy of Medical Science (Approval number: DWSP20240309). All experiments involving live CHIKV were conducted at the ABSL‐3 laboratory of the National Kunming High‐level Biosafety Primate Research Center.

## Consent

The authors have nothing to report.

## Conflicts of Interest

The authors declare no conflicts of interest.

## Supporting information




**Supporting File 1**: mco271138‐sup‐0001‐SuppMat.docx.

## Data Availability

All the datasets generated and/or analyzed during the current study are available in the NCBI repository under accession numbers PRJNA1288148 (Metagenome), MetaboLights under accession numbers MTBLS12856 (Metabolomics), and iproX under accession number IPX0013007000 (Proteomics).
