# Supplementary material for: Parenteral CHIKV mRNA Vaccination Reshapes the Gut Microbial, Metabolic, and Immune Landscapes
Source: MedComm (2020). 2026 Nov 8;7(11):e71138. doi: 10.1002/mco2.71138 (PMC13744653; doi:10.1002/mco2.71138)
Supplement: Supplementary file 1 [file MCO2-7-e71138-s001.docx]

***Supplementary materials for***

**Parenteral CHIKV mRNA Vaccination Reshapes the Gut Microbial, Metabolic, and Immune Landscapes**

**Authors:** Hongyu Chen^1#^, Wenhao Xie^1#^, Yun Yang^1#^, Cong Tang^1#^, Xiaoming Liang^1^, Yuxia Yuan^1^, Junbin Wang^1^, Yanan Zhou^1^, Hao Yang^1^, Qing Huang^1^, Wenqi Quan^1^, Wenhai Yu^1^, Haixuan Wang^1^, Daoju Wu^1^, Youchun Wang^1,2,3*^, Shuaiyao Lu^1,2,3*^

**Affiliations:** ^1^State Key Laboratory of Respiratory Health and Multimorbidity, Institute of Medical Biology, Chinese Academy of Medical Sciences & Peking Union Medical College (IMBCAMS&PUMC), Kunming, Yunnan China, 650118; ^2^Key Laboratory of Pathogen Infection Prevention and Control (Peking Union Medical College), Ministry of Education, Beijing, China; ^3^Yunnan Key Laboratory of Cross-Border Infectious Disease Control and Prevention and Novel Drug Development, Kunming, China

#These authors contribute equally.

***Correspondence**

Shuaiyao Lu, DVM, PhD, Institute of Medical Biology, Chinese Academy of Medical Sciences & Peking Union Medical College, 935 Jiaoling Road, Kunming, Yunnan China 650118, Email: lushuaiyao-km@163.com.

Youchun Wang, Institute of Medical Biology, Chinese Academy of Medical Sciences & Peking Union Medical College, 935 Jiaoling Road, Kunming, Yunnan China 650118, Email: wangyc@nifdc.org.cn.

**Supplementary Figure 1**

(**A**) Alpha diversity of fecal microbiota. Sobs index in each group of fecal microbes (Top), and Shannon index in each group of fecal microbes (Bottom). Kruskal–Wallis test (Dunn’s post hoc) with FDR

(**B**) Principal coordinate analysis (PCoA) of fecal microbiota based on metagenomic sequencing at baseline (BG), each vaccination group (S1, S2) and (**C**) infection group (CHIKV-Ctrl). Statistical differences among clusters were analyzed using permutational analysis of variance (PERMMANOVA). R^2^ was used to interpret the degree of difference among each group.

(**D**) Heatmap of the abundance(Top30) of Fecal microbiota (genus level) in V1(left panel) and V2(right panel) group.

(**E**) Heatmap of the abundance of Fecal microbiota (genus level) in V1(left panel), V2(right panel) and infection group.

(**F**) Comparative statistical analysis of abundance of key microbes (identified by LEfSe) between each groups. (Kruskal–Wallis test (Dunn’s post hoc) with FDR; *q < 0.05, ** q < 0.01).

**Supplementary Figure 2**

Dynamics of serum immunoregulatory (**A**) and inflammatory cytokines(**B**) in the vaccination and infected groups. Colour boxes indicate different time periods. Green: phase of first immunisation; phase of yellow: second immunization; phase of blue: viral infection.

**Supplementary Figure 3**

Principal coordinate analysis (PCoA) of fecal microbiota based on metagenomic sequencing of Vaccine group1(**A**) and vaccine group 2(**B**), before and after infection. Statistical differences among clusters were analyzed using permutational analysis of variance (PERMMANOVA). R^2^ was used to interpret the degree of difference among each group.

(**C**) Alpha diversity of fecal microbiota. Sobs index in each group of fecal microbes (left panel), and Shannon index(right panel) in each group of fecal microbes (Bottom). (Kruskal–Wallis test (Dunn’s post hoc) with FDR.

(**D**) PCoA of fecal microbiota based on metagenomic sequencing of immunized-infected group(CV1,CV2) and infection group (CHIKV-Ctrl). Statistical differences among clusters were analyzed using permutational analysis of variance (PERMMANOVA). R^2^ was used to interpret the degree of difference among each group.

(**E**) Comparative statistical analysis of abundance of key microbes between each groups. Kruskal–Wallis test (Dunn’s post hoc) with FDR.

(**F**) Heatmap of the relative abundances of virulence factors of microbes in fecal of each group. The relative abundance is indicated by the color scale bar on the left.

**Supplementary Figure** 4

(**A**) Alpha diversity of GI microbiota. Sobs index in each group of GI microbes (Top panel), and Shannon index (Bottom panel) in each group of fecal microbes. (Kruskal–Wallis test (Dunn’s post hoc) with FDR.

(**B**) PCoA of colon (Top panel) and rectum (Bottom panel) microbiota based on metagenomic sequencing of immunized-infected group(CV1,CV2) and infection group (CHIKV-Ctrl). Statistical differences among clusters were analyzed using permutational analysis of variance (PERMMANOVA). R^2^ was used to interpret the degree of difference among each group.

(**C**) Composition and structure of colon and rectum intestinal microbiota (genus level) in both groups.

**Supplementary Figure 5**

**(A)** PLS-DA of fecal metabolites before and after vaccination. Group differences analyzed via PERMANOVA; R^2^ was used to interpret the degree of difference among each group.

(**B**) The metabolites at the DEPs for variable importance (VIP) analysis, data were normalized by Z-score standardization method.

(**C**) Time-series analysis of changes in intestinal metabolite abundance following vaccine 1(Top panel) and vaccine 2 (Bottom panel) immunization.

(**D**) Metabolites whose expression was upregulated after immunisation with both vaccines were selected (Top panel) for KEGG functional enrichment (Bottom panel)in the time series analysis.

(**E**) All annotated metabolites were subjected to correlation matrix analysis with key microorganisms (Top panel), and metabolites significantly and positively correlated with *S. copri* were selected for functional enrichment analysis (Bottom panel).

(**F**) Correlation matrix analysis of metabolites in purine metabolic pathways with key microorganisms.

**Supplementary Figure 6**

Comparison of cytokines between CV and C groups. Statistical tests: one-way ANOVA and post hoc *Scheffe test*, FDR corrected (*q < 0.05).

**Supplementary Figure 7**

(**A**)Immunofluorescence staining of CD4^+^ T cell in gut tissues (Top panel). Quantification was performed using ImageJ; representative images are shown (Bottom panel). Statistical comparisons were analyzed using one-way ANOVA and post hoc *Scheffe test* with FDR correction (**q* < 0.05).

(B)AB-PAS staining of goblet cell in gut tissues (Top panel). Quantification was performed using ImageJ; representative images are shown (Bottom panel). Statistical comparisons were analyzed using one-way ANOVA and post hoc *Scheffe test* with FDR correction (**q* < 0.05).

**Supplementary Figure 8**

Proposed integrative model of CHIKV mRNA vaccination-associated intestinal remodeling and protective gut immunity. Parenteral CHIKV mRNA vaccination induces coordinated remodeling of the gut microenvironment, including shifts in microbial composition, purine- and bile acid-related metabolism, and selected cytokine responses. These changes partially resemble intestinal ecological alterations observed during active CHIKV infection, but they should not be interpreted as equivalent to infection-associated pathology. In contrast to active infection, vaccination occurs in the absence of sustained viral replication and establishes a primed intestinal immune state before challenge. Upon CHIKV exposure, this immune priming is associated with reduced intestinal viral burden, attenuated tissue injury, preserved epithelial barrier integrity, and enhanced antigen-presentation and immunoglobulin-related responses.

**Supplementary Table S1. Experimental groups, Number of animals, Sample types, and corresponding analyses**

| **Experimental stage** | **Group** | **N** | **Experimental procedure** | **Sample type** | **Main analyses** |
| --- | --- | --- | --- | --- | --- |
| Baseline before vaccination | Ctrl_BG | 3 | PBS control before immunization | Feces and serum | Fecal metagenomics, fecal metabolomics, Olink cytokine profiling |
| Baseline before vaccination | V1BG | 3 | Before V1 immunization | Feces and serum | Fecal metagenomics, fecal metabolomics, Olink cytokine profiling |
| Baseline before vaccination | V2BG | 3 | Before V2 immunization | Feces and serum | Fecal metagenomics, fecal metabolomics, Olink cytokine profiling |
| After primary vaccination | V1_S1 | 3 | First intramuscular immunization with V1 | Feces and serum | Fecal metagenomics, fecal metabolomics, Olink cytokine profiling |
| After primary vaccination | V2_S1 | 3 | First intramuscular immunization with V2 | Feces and serum | Fecal metagenomics, fecal metabolomics, Olink cytokine profiling |
| After booster vaccination | V1_S2 | 3 | Second intramuscular immunization with V1 | Feces and serum | Fecal metagenomics, fecal metabolomics, Olink cytokine profiling |
| After booster vaccination | V2_S2 | 3 | Second intramuscular immunization with V2 | Feces and serum | Fecal metagenomics, fecal metabolomics, Olink cytokine profiling |
| Before CHIKV challenge | V1 | 3 | Completed two-dose V1 immunization | Feces and serum | Fecal metagenomics, fecal metabolomics, Olink cytokine profiling |
| Before CHIKV challenge | V2 | 3 | Completed two-dose V2 immunization | Feces and serum | Fecal metagenomics, fecal metabolomics, Olink cytokine profiling |
| After CHIKV challenge | CV1 | 3 | V1-immunized animals challenged intramuscularly with CHIKV | Feces and serum | Longitudinal fecal metagenomics and Olink cytokine profiling |
| After CHIKV challenge | CV2 | 3 | V2-immunized animals challenged intramuscularly with CHIKV | Feces and serum | Longitudinal fecal metagenomics and Olink cytokine profiling |
| After CHIKV challenge | CHIKV-Ctrl | 3 | PBS-treated control animals challenged intramuscularly with CHIKV | Feces and serum | Longitudinal fecal metagenomics, Olink cytokine profiling, and viral-load measurement |
| Terminal collection | CV1 | 3 | V1-immunized and CHIKV-challenged animals | Blood, small- and large-intestinal contents, gastrointestinal tissues, Peyer’s patches, and mesenteric lymph nodes | Viral-load measurement, intestinal-content metagenomics, untargeted metabolomics, histopathology, immunofluorescence, cytokine profiling, and tissue proteomics |
| Terminal collection | CV2 | 3 | V2-immunized and CHIKV-challenged animals | Blood, small- and large-intestinal contents, gastrointestinal tissues, Peyer’s patches, and mesenteric lymph nodes | Viral-load measurement, intestinal-content metagenomics, untargeted metabolomics, histopathology, immunofluorescence, cytokine profiling, and tissue proteomics |
| Terminal collection | CHIKV-Ctrl | 3 | Unvaccinated and CHIKV-challenged animals | Blood, small- and large-intestinal contents, gastrointestinal tissues, Peyer’s patches, and mesenteric lymph nodes | Viral-load measurement, intestinal-content metagenomics, untargeted metabolomics, histopathology, immunofluorescence, cytokine profiling, and tissue proteomics |

**Abbreviations:** Ctrl_BG, baseline samples from the PBS-treated control group; V1BG and V2BG, baseline samples collected before V1 or V2 immunization; S1, samples collected after the primary immunization; S2, samples collected after the booster immunization; CV1 and CV2, V1- or V2-immunized animals following CHIKV challenge; CHIKV-Ctrl, unvaccinated animals following CHIKV challenge;
